# Supplementary material for: Enhanced Immunogenicity of Engineered HER2 Antigens Potentiates Antitumor Immune Responses
Source: Vaccines (Basel). 2020 Jul 22;8(3):403. doi: 10.3390/vaccines8030403 (PMC7563373; doi:10.3390/vaccines8030403)
Supplement: Supplementary file 1 [file vaccines-08-00403-s001.pdf]

Supplementary Information

Supplementary Figure S1. The expression of HER2 on THP-1

Supplementary Figure 2. The expression of HER2 on mouse B cells and monocytes.

Supplementary Figure 3. The phosphorylation of Erk on the engineered HER2 transduced THP-1.

Supplementary Figure 3. The live population of mouse BVAC.

Supplementary Figure 4. The expression of PD-L1 on tumor cells and the secretion of INF-γ by CD8+ T cells.

Supplementary Figures

Supplementary Figure S1. The expression of HER2 on THP-1 and phosphorylation of HER2 signaling molecule.

Human monocytic cell-line THP-1 cells were transduced with 100 MOI of adK684, adK965, and adK1117 and the expression of their antigen was analyzed by flow cytometry. (A) The geometric mean of live/dead gated $HER2^{+}$ cells. The live gated population of each adenoviral vector transduced THP-1 cells (B and C). (D) The phosphorylation of downstream signaling molecules of HER2 was analyzed by immunoblot assay. All data are representative of two independent experiments. (*P<0.05, **P<0.01, ***P<0.001).

Supplementary Figure S2. The expression of HER2 on mouse B cells and monocytes.

(A) The expression of HER2 on mouse BVACs. B220+ and CD11b+ splenocytes were isolated from naïve BALB/c mice by micro beads and transduced with 200 MOI of adenovirus vectors carrying each antigen.

Supplementary Figure S3. The phosphorylation of Erk on the engineered HER2 transduced THP-1.

THP-1 cells were transduced with adenovirus coding for each engineered HER2 antigen and analyzed phosphorylation of Erk 24hours after transduction.

Supplementary Figure S4. The live population of mouse BVAC.

B cells and monocytes was isolated from naïve BALB/c mice and transduced 200 MOI of each adenoviral vectors. After 18 hours, the BVAC was washed and analyzed the live and dead cells with flow cytometry. (A and B)

Supplementary Figure S5. The expression of PD-L1 on tumor cells and the secretion of INF-γ by CD8+ T cells.

A total of 5 x 10^5 cells of HER2 expressing CT26 was inoculated naïve BALB/c mice and immunized 10 days after tumor inoculation. On day 17, tumor infiltrating lymphocytes were isolated and analyzed (A). The tumor infiltrating lymphocytes were stimulated with HER2 epitope (hP63) and analyzed the secretion of IFN-g by CD8+ T cells were analyzed by flow cytometry (B and C). The expression of PD-L1 on HER2+ CD45.2- CT26/HER2 tumor cells was analyzed (D and E). (*P<0.05, **P<0.01, ***P<0.001).

Supplementary method

Immunoblot analysis

Cytoplasmic fractions of cells were prepared as follows. THP-1 cells were transduced with each indicated adenoviral vectors and washed 4 hours after transduction. After 12 and 24 hours after viral transduction, THP-1 cells were washed once with ice-cold PBS and collected by centrifugation at 5000 r.p.m. for 10 minutes. The cells were resuspended in RIPA buffer (R0278, Sigma-Aldrich) with 1 mmol/L DTT and 0.25 mmol/L PMSF and a proteinase inhibitor cocktail (535140, Calbiochem). The mixtures were vortexed and incubated in the ice for 30 minutes. The cytoplasmic extracts in the supernatant were collected after centrifugation at 13,000 r.p.m. for 10 minutes. These cell extracts were loaded onto SDS-PAGE gels and then transferred to PVDF membranes using iBlot 2 PVDF Mini Stacks (Invitrogen). The membranes were stained with specific antibodies, and chemiluminescence was visualized using an LAS-3000 Lumino-Image analyzer (Fuji film).

Preparation of tumor-infiltrating lymphocytes

Tumor tissues from CT26/HER2 tumor bearing mice were homogenized and dissociated with gentle MACS Dissociator (Miltenyi Biotec). The dissociated tumor tissues were digested in 2% FBS RPMI medium containing 1 mg/mL collagenase D (Roche), 100 mg/mL hyaluronidase (Sigma-Aldrich), and 100 mg/mL DNase I (Sigma-Aldrich) at 37℃ for 1 hour. The lymphocytes were isolated from the digested tissues, by 40% and 60% discontinuous Percoll (GE Healthcare) gradient centrifugation.

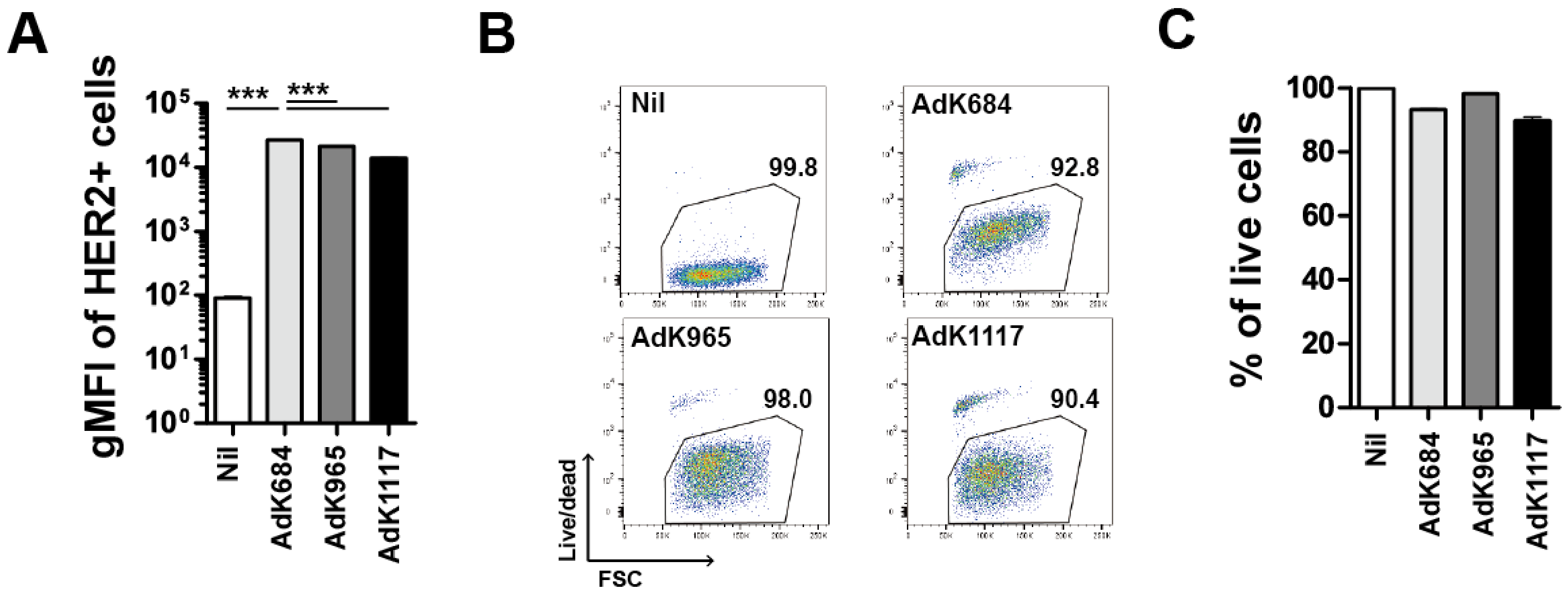

Supplementary Figure 1. Jeon et al.

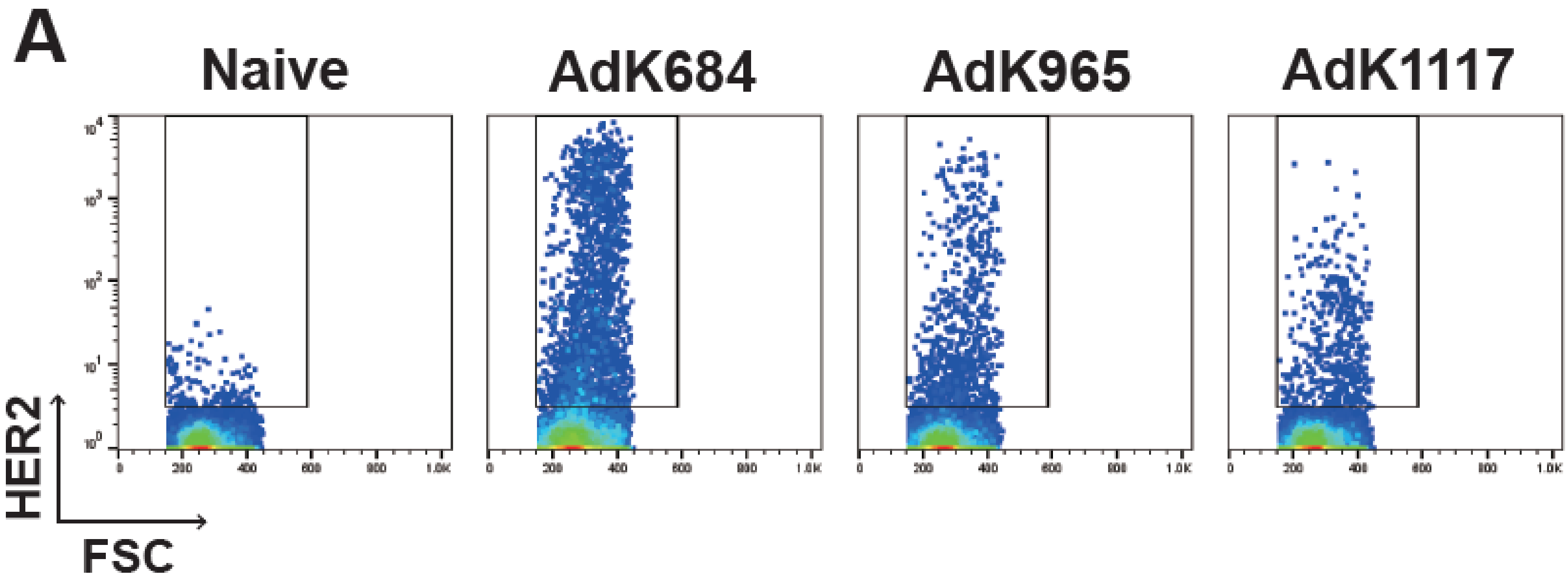

Supplementary Figure 2. Jeon et al.

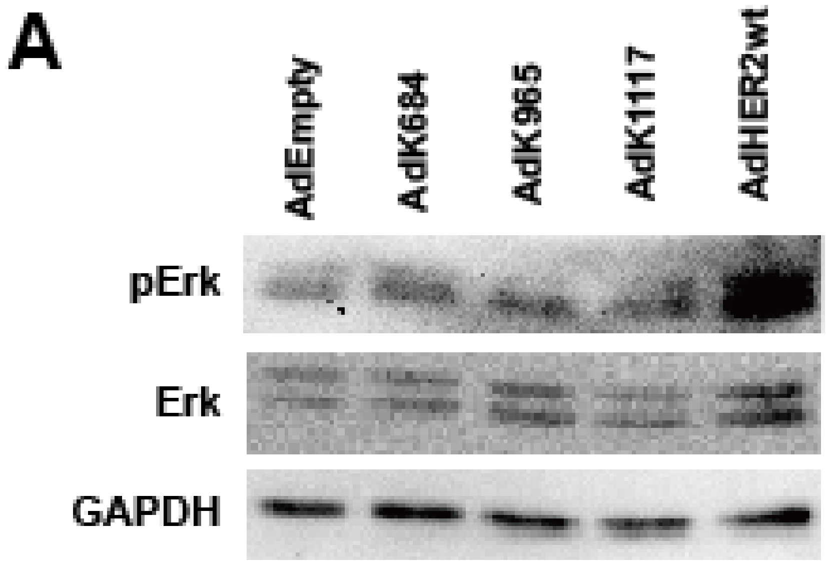

Supplementary Figure 3. Jeon et al.

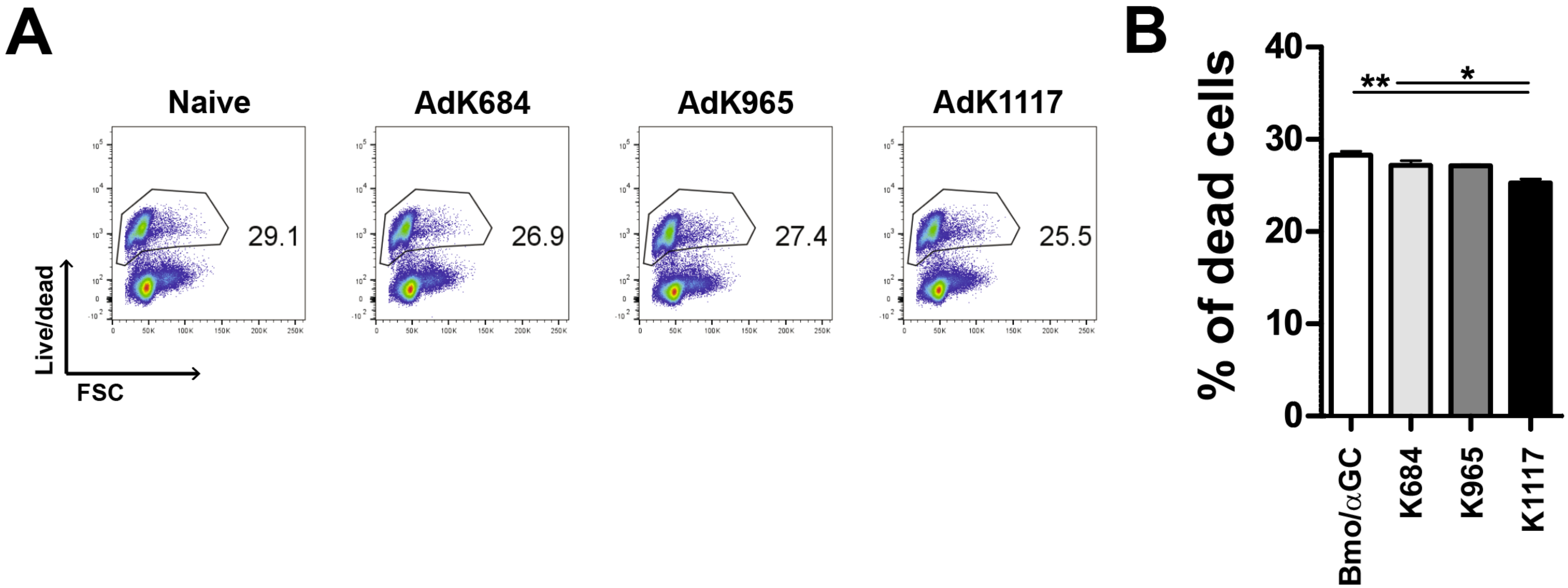

Supplementary Figure 4. Jeon et al.

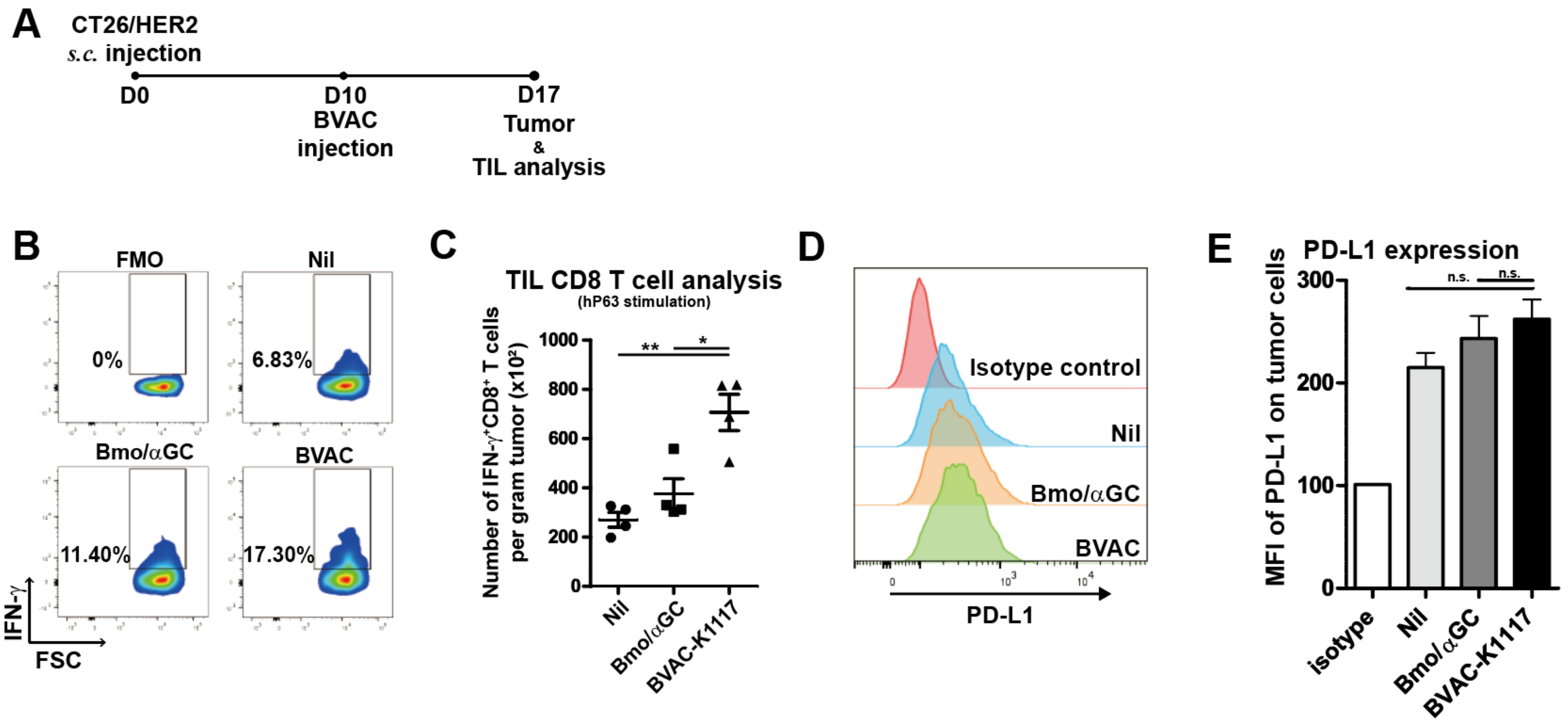

Supplementary Figure 5. Jeon et al.
